# Blood Viscosity and the Expression of Inflammatory and Adhesion Markers in Homozygous Sickle Cell Disease Subjects with Chronic Leg Ulcers

**DOI:** 10.1371/journal.pone.0068929

**Published:** 2013-07-26

**Authors:** Andre S. Bowers, Harvey L. Reid, Andre Greenidge, Clive Landis, Marvin Reid

**Affiliations:** 1 Department of Basic Medical Sciences (Physiology Section), University of the West Indies, Mona, Kingston 7, Jamaica; 2 Edmund Cohen Laboratory for Vascular Research, Chronic Disease Research Centre, University of the West Indies, Cave Hill Campus, Saint Michael, Barbados; 3 Sickle Cell Unit, Tropical Medicine Research Institute, University of the West Indies, Mona, Kingston 7, Jamaica; University of Illinois at Chicago, United States of America

## Abstract

**Objective:**

To determine differences in TNF-α, IL-1β, IL-10, sICAM-1 concentrations, leg hypoxia and whole blood viscosity (WBV) at shear rates of 46 sec^-1^ and 230 sec^-1^ in persons with homozygous S sickle cell disease (SCD) with and without chronic leg ulceration and in AA genotype controls.

**Design:**

**& Methods**: fifty-five age-matched participants were recruited into the study: 31 SS subjects without leg ulcers (SS_n_), 24 SS subjects with leg ulcers (SS_u_) and 18 AA controls. Haematological indices were measured using an AC.Tron Coulter Counter. Quantification of inflammatory, anti-inflammatory and adhesion molecules was performed by ELISA. Measurement of whole blood viscosity was done using a Wells Brookfield cone-plate viscometer. Quantification of microvascular tissue oxygenation was done by Visible Lightguide spectrophotometry.

**Results:**

TNF-α and whole blood viscosity at 46 sec^-1^ and 230 sec^-1^ (1.75, 2.02 vs. 0.83, 1.26, *p*<0.05) were significantly greater in sickle cell disease subjects than in controls. There were no differences in plasma concentration of sICAM-1, IL-1β and IL-10 between SCD subjects and controls. IL-1β (median, IQR: 0.96, 1.7 vs. 0, 0.87; *p*<0.01) and sICAM-1 (226.5, 156.48 vs. 107.63, 121.5, *p*<0.005) were significantly greater in SS_u_ group compared with SS_n_. However there were no differences in TNF-α (2, 3.98 vs. 0, 2.66) and IL-10 (13.34, 5.95 vs. 11.92, 2.99) concentrations between SS_u_ and SS_n_. WBV in the SS_u_ group at 46 sec^-1^ and at 230 Sec 1 were 1.9 (95%CI; 1.2, 3.1) and 2.3 (1.2, 4.4) times greater than in the SS_n_ group. There were no differences in the degree of tissue hypoxia as determined by lightguide spectrophotometry.

**Conclusion:**

Inflammatory, adhesion markers and WBV may be associated with leg ulceration in sickle cell disease by way of inflammation-mediated vasoocclusion/vasoconstriction. Impaired skin oxygenation does not appear to be associated with chronic ulcers in these subjects with sickle cell disease.

## Introduction

Chronic leg ulceration is the most common cutaneous manifestation of homozygous sickle cell disease (SCD) [1], predominantly affecting the medial and lateral malleoli, and to a lesser extent, the anterior shin or dorsum of the foot [2]. A cumulative involvement of about 70% has been reported by the 30^th^ year of life, establishing these lesions as a major source of morbidity among Jamaican SCD patients [3]. However, the most recent estimates among this group of patients have reported a prevalence of 29.5% and a cumulative incidence of 16.7% [4]. The tropical climate and low socio-economic status are likely contributors to the aetiology of chronic leg ulcers in the Jamaican population [4]. Other risk factors for ulceration include high white cell count, serum lactate dehydrogenase and venous incompetence [5,6].

The propensity of sickle red blood cells (RBC) for vasoocclusion and abnormal flow behaviours are central in propagating some of the diverse vascular symptoms associated with the condition, including systemic [7] and pulmonary [7–9] hypertension. Endothelial dysfunction is a feature of sickle cell disease [10,11] which likely influences whole blood viscosity and blood flow. A possible involvement of incompetent calf veins and elevated intravascular pressures in sickle ulceration, especially in the dependent position [5,6], suggest a role of abnormal flow behaviours in its development and/or maintenance. Moreover, a preponderance of abnormal adhesion properties [7–9] in SCD exacerbates this low flow state thereby influencing haemoglobin S polymerization, relative hyperviscosity, ischaemia and reperfusion tissue injury. The adhesion molecule sICAM-1 is constitutively expressed by endothelial cells and is up-regulated in response to inflammatory stimulus such as the cytokines TNF-α and IL-1β [12]. Shiu et al. demonstrated an increase in both membrane bound and soluble sICAM-1 expression upon perfusion of endothelial cells with sickle erythrocytes [13], where there was a greater concentration of inflammatory mediators suggesting a mechanistic link between vascular inflammation and adhesion. In addition, SCD is associated with increased propensity to infections, possibly a consequence of reticuloendothelial dysfunction. Infection-mediated endothelial activation by way of NF*k*-β nuclear translocation is important in the inflammatory response through the synthesis and secretion of pro-inflammatory cytokines [14,15], a correlate of clinical severity in SCD [16,17]. However, whether these inflammatory markers are associated with leg ulceration in sickle cell disease is unclear. Abnormal rheological properties of sickle cell disease characterized by an abnormal viscosity profile may be linked to a pro-adhesive state in the microcirculation. Reduced tissue perfusion has been reported in Jamaican ulcer patients [5] and could be related to viscosity changes.

The investigation of microvascular cutaneous blood flow has been used extensively in the assessment of vascular abnormalities in diseases such as diabetes [18,19] and sickle cell disease [6,20,21]. Laser Doppler fluxmetry and venous occlusion plethysmography are among the established noninvasive methods of quantifying microcirculatory blood flow. Visible lightguide spectrophotometry is another noninvasive measure which has been widely used in the assessment of amputation viability in critically ischaemic limbs [22–24] and lower limb cutaneous perfusion in diabetes [25] but remains unexplored in sickle cell disease. Spectrophotometry in the visible range has been developed for the determination of oxygenation in the inflamed skin [23]. Given the ubiquity of mediators of abnormal blood flow in SS disease, we decided to investigate the degree of tissue hypoxia in subjects with HbSS in order to ascertain whether tissue ischaemia may be implicated in sickle cell leg ulcers.

We hypothesize that cutaneous leg ulceration in HbSS is associated with an inflammatory aetiology marked by the up-regulation of pro-inflammatory cytokines and vascular adhesion molecules. In the present study the haematocrit-viscosity ratio (HVR), a measure of the effectiveness of erythrocytes in transporting oxygen [26–29], was used to assess blood flow conditions in the sickle cell disease subjects with ulcers and SCD controls. Additionally, cutaneous microvascular oxygenation was determined by visible lightguide spectrophotometry. We propose that abnormal rheology, inflammation and endothelial dysfunction have important roles in the pathogenesis of chronic leg ulceration in homozygous sickle cell disease.

## Materials and Methods

A descriptive, cross-sectional study was conducted at the Sickle Cell Unit (SCU), Tropical Medicine Research Institute, University of the West Indies (UWI), Mona and the Department of Basic Medical Sciences (Physiology Section), UWI, Mona.

### Ethics Statement

Ethical approval was granted by the University Hospital of the West Indies/Faculty of Medical Sciences/University of the West Indies (UHWI/FMS/UWI) Ethics Committee. The study was performed in accordance with the Declaration of Helsinki. Volunteers gave written, informed consent and completed an interviewer administered questionnaire.

### Subjects

Fifty five subjects with homozygous sickle cell disease and 18 AA controls were identified and recruited at the SCU, UWI, Mona. Twenty four of the volunteers had an active ulcer at the time of the study and 31 had no history of ulceration. Twenty seven of the subjects with SCD, 11 with and 16 without active ulcers, participated in skin perfusion studies. Subjects were studied in the steady sate to exclude any possible effects caused by sickle painful crisis-related alterations in haemorheological and inflammatory markers. Steady state was defined as no sickle related event within the 4 weeks or blood transfusion within 3 months of the experimental study [6].

### Sample collection

Venous blood (10 mL) was drawn from an antecubital vein into potassium EDTA-anticoagulated (1.5 mg/mL) vacutainer tubes. Five mL of whole blood were stored at room temperature (25°C) for viscometry and haematological analysis. The remaining 5 mL were centrifuged at 1000 g within 30 minutes of collection. Plasma aliquots were then stored into 1.5 mL Eppendorf tubes at -20°C for use in ELISA determinations.

### Haematological analysis

Five ml of venous blood were drawn from an arm vein into EDTA anti-coagulated vacutainer tubes. Measurement of red blood cell, platelet counts and haemoglobin concentration were done using an AC.Tron Coulter Counter with Act.Diff Pak 4C controls (Coulter Electronics, Hialeah, FL, USA). The indices measured were haemoglobin concentration (Hb) (g/dL), red blood cell concentration (RBC) (x10^12^ cells/L), haematocrit (Hct) (%), platelet count (Plt) (x10^9^/L), white cell count (WBC) (x10^9^/L), mean corpuscular volume (MCV) (fL), mean corpuscular haemoglobin (MCH) (pg), mean corpuscular haemoglobin concentration (MCHC) (%) and red cell distribution width (RDW).

### Cytokine assay

The concentrations of IL-1β, TNF-α, IL-10 and sICAM-1 in the circulation of all participants were determined by sandwich enzyme-linked immunosorbent assay (R&D Systems, 614 McKinley Place NE, Minneapolis, MN, USA).

### Viscometry

Whole blood viscosity was measured with a Wells Brookfield cone-plate viscometer at a low and a high shear rate of 46 sec^-1^ and 230 sec^-1^ respectively. Measurements were performed within 15 minutes to an hour from time of sampling at native haematocrit at 37°C. Haemorheological determinations conformed to standard procedures for haematological and viscometric procedures [30].

### Haematocrit-viscosity ratio (HVR)

The HVR was calculated as the ratio of the native haematocrit to whole blood viscosity [26,27,29,31].

### Microvascular perfusion studies of the lower leg

Determination of microvascular tissue oxygenation (SO_2_) was performed on 27 subjects with HbSS, 16 with active ulcers at time of study and 11 subjects without ulcers using a Visible Lightguide Spectrophotometer (RM200 S02 Monitor, Whitland Research, Whitland, UK). The optical determination of skin oxygenation by lightguide spectrophotometry (O2C) has been described in previous studies [25,32]. The instrument functions by detecting the ratio of haemoglobin: oxyhaemoglobin in the microvasculature.

The Visible Lightguide Spectrophotometer was set up in a temperature controlled environment (24°C) free from the presence of fluorescent lighting. The machine was then calibrated using readings at the opposite ends of the colour spectrum (Black and White). Participants were then asked to sit on the examination bed with their feet positioned such that the shin was parallel to the floor. The probe was then lightly placed on the leg, 3cm medial to the shin and an initial So_2_ reading was obtained. The probe was then moved down the leg at 1 cm intervals, down to the ankle.

In determining the degree of hypoxia, critical ischaemia was defined when 15% or more of the measured values fell below an oxygen saturation threshold of 15% SO_2_.

### Statistical analysis

Data were presented as median (inter-quartile range) or means (standard deviations). Differences in median values per group were determined by the Kruskal-Wallis equality-of-populations rank test: SCD vs. AA; SS_n_ vs. SS_u_. Regression analysis was used to test for relationships between independent variables and leg ulceration. Data were analyzed using Stata Statistics Data Analysis *v*10.1 (Statacorp, College Station, Texas).

## Results

There were no differences in the anthropometric variables (age, height, weight, body mass index) and LDH by genotype. As expected, subjects with sickle cell disease had significantly lower haematocrit, haemoglobin concentrations and RBC concentrations but significantly increased leucocyte counts, platelet concentrations and RDW (Table 1). However there was no difference in MCV value between AA and subjects with sickle cell disease.

**Table 1:** Anthropometric variables, haematological indices and lactate dehydrogenase concentrations in sickle cell disease patients and control group.

**Variables**	**AA (n = 18)**	**SS (n = 55)**
**Age (yrs)**	34.31±9.7	34.84±10.4
**Height (cm)**	168.1±8.3	163±21.2
**Weight (Kg)**	68.7±12.5	60.5±9.7
**Body mass index**	24.23±3.57	24.04±8.43
**LDH (IU/L)**	(n = 12)378±172.02	(n = 48)1216.62±403.8
**Hb (g/dL)**	13.1 (2.7); 10.4, 15.7	7.9 (2.2); 4.9, 10.6*
**Hct (%)**	40.8 (8.2); 33, 49.8	23.7 (6.5); 15.5, 32.5*
**RBC (x10^12^ cells/µL)**	4.7 (1.3); 3.6, 5.7	2.7 (0.8); 1.6, 4.2*
**MCV (fL)**	88 (7.6); 63.3, 101	88 (8.2); 71.6, 103
**MCH (pg)**	28.9 (1.7); 18.6, 33.1	29.3 (3.4); 21.7, 34.8
**MCHC (%)**	32.2 (1.8); 29.5, 35.3	33.1 (22.4);21, 37.2*
**Plt (x10^9^/L)**	264.5(127); 171, 451	386 (151);185, 746*
**WBC (x10^9^/L)**	5.4 (1.6); 3.6, 9.6	11 (3.9);5.1, 37.8*
**RDW**	12.9 (1.3); 11.1, 16.8	22.3 (4.5);14.5; 33.9*

Anthropometric and LDH values are mean±SD; haematological values are median(inter-quartile range) minimum value, maximum value. * significant difference at p<0.05.

The mean age, weight and BMI of subjects with sickle disease and leg ulcers, SS_u_, were significantly greater than subjects with sickle cell disease without leg ulcers, SS_n_. However there was no difference in mean height between the SS_n_ and SS_u_ groups (Table 2). Haematocrit, MCV and RBC counts were significantly lower in the SS_u_ group compared with SS_n_ group. In contrast, there were no significant difference between SS_u_ group and the SS_n_ for other haematological variables and LDH (Table 2).

**Table 2:** Anthropometric variables, haematological variables and lactate dehydrogenase concentrations in sickle cell disease patients with chronic leg ulcers and patients without ulcers.

**Variables**	**SS_n_ (n= 31)**	**SS_u_ (n= 24)**
**Age (yrs)**	31.6±10.1	38.7±9.7*
**Height (cm)**	162±19.6; 111, 185.7	163.6±23.4; 111, 191.9
**Weight (Kg)**	58.1±10.5; 44, 98.9	63.6±7.7; 50, 82.7*
**Body mass index**	23.29±9.17; 16.48, 51.75	25.0±7.44; 17.87, 44.23*
**LDH (IU/L)**	(n=29)1258.71±431.5; 545, 2129	(n= 19) 1154.58±361.36;612, 1970
**Hb (g/dL)**	7.9 (2.1); 6.3, 10.6	7.6 (2.3); 4.9, 10.4
**Hct (%)**	24.7 (6.2); 18.9, 32	22.8 (6.6); 15.5, 32.5*
**RBC (x10^12^ cells/µL)**	2.8 (0.7); 1.9, 4.2	2.5 (0.8); 1.6, 3.8*
**MCV (fL)**	87 (6.4); 71.6, 103	91 (7.5); 80.8, 97.4*
**MCH (pg)**	28.8(3); 21.7, 34.8	29.6 (3.2); 26.2, 34.8
**MCHC (%)**	33 (2.5); 21, 37	33.2 (2.5); 31.4, 37.2
**Plt (x10^9^/L)**	409 (159); 185, 645	351.5(156); 246, 746
**WBC (x10^9^/L)**	11 (4.6); 5.1, 37.8	11.2 (3.9); 7.9, 19.2
**RDW**	21.6 (4.6); 14.5, 33.9	23.1 (4.1); 17, 30.2

Anthropometric and LDH values are mean±SD; haematological values are median(inter-quartile range) minimum value, maximum value. * significant difference at p<0.05.

Plasma concentrations of the pro-inflammatory cytokines TNF-α and IL-1β, the adhesion molecule sICAM-1 and the anti-inflammatory cytokine IL-10 were measured in a total of 55 adult subjects with sickle cell disease and 17 haemoglobin AA controls. There were 32 males and 23 females with the SS genotype (median age 32; range 18-55). Of the SS subjects, 24 had an active ulcer (7 females & 17 males; median age 36.5; range 21-54) at the time of study (SS_u_) and 31 (16 females & 15 males; median age 32; range 18-55) were asymptomatic, HbSS subjects without ulcers (SS_n_).

Median TNF-α (*p* = 0.001) concentration was significantly increased in the sickle cell disease group. However, there were no differences in ICAM-1, IL-1β and IL-10 concentrations between patients with sickle cell disease and the control group (Table 3).

**Table 3:** Median inflammatory, anti-inflammatory and adhesion cytokine concentrations in sickle cell disease patients and AA controls.

**Variables**	**AA (n = 17)**	**SS (n = 55)**
**TNF-α (pg/mL)**	0(0); 0 7.2	0.63 (3.90); 0, 15.25*
**IL-1β (pg/mL)**	0 (0.05); 0, 3.3	0 (0.91); 0, 6.33
**ICAM-1 (ng/mL)**	150.05 (89.91); 0, 249.01	93.81 (209.28); 0, 445.01
**IL-10 (ng/mL)**	6.36 (3.06); 0, 11.09	9.12 (12.76); 0, 33.56

Values are media(inter-quartile range) minimum, maximum value. * significant difference at *p*<0.05.

Median TNF-α (*p* = 0.001) concentration was significantly increased in the sickle cell disease group. Of the 24 subjects with active ulcers, TNF-α was detectable in 18 and IL-1β was detectable in 14 participants. The frequency of cytokine detection in sickle cell disease patients without ulcers was lower in comparison to the ulcer group, which showed 12 of 31 presenting with detectable concentrations of TNF-α and 10 with IL-1β.

**Figure 1:**
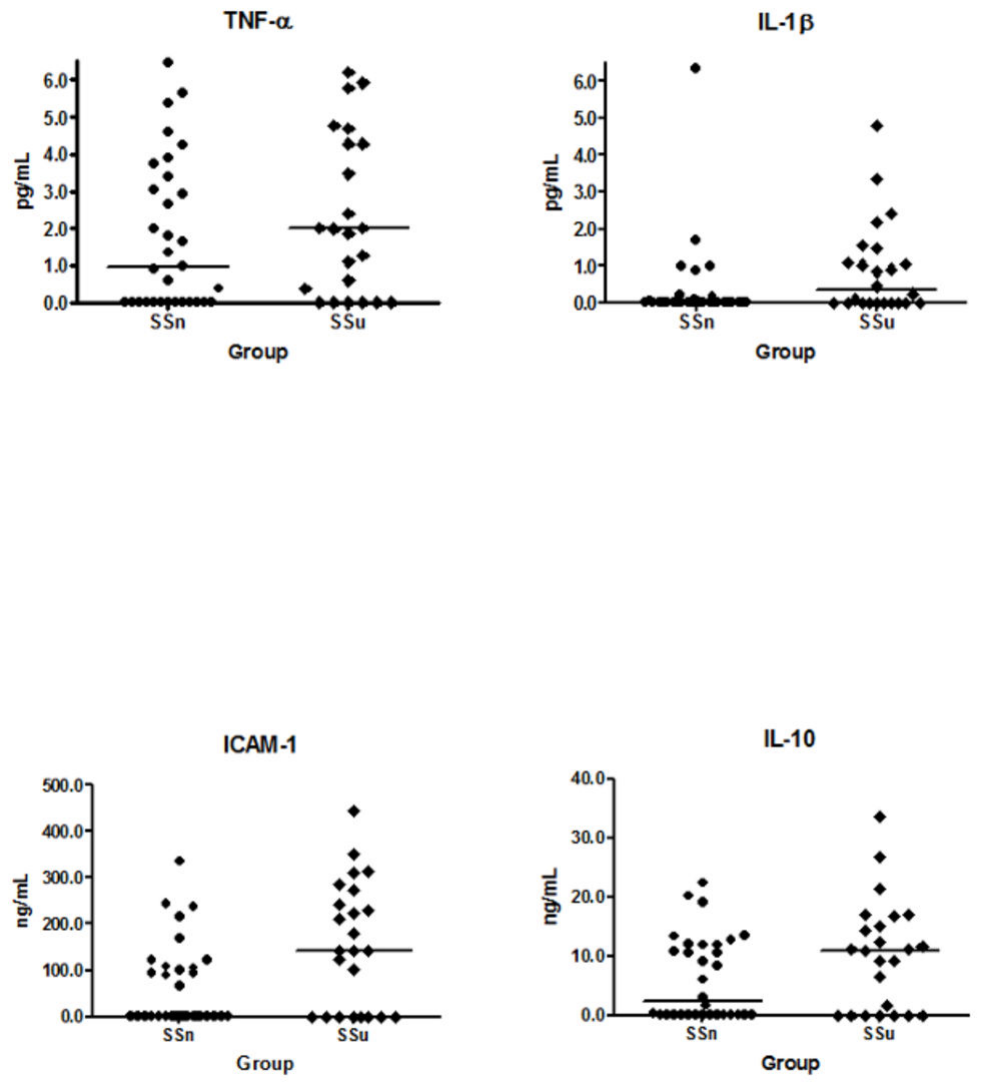
Plasma concentration distributions of interleukin-1beta, tumor necrosis factor-alpha, inter-cellular adhesion molecule-1 and interleukin-10 in SCD patients with ulcers and without ulcers. IL-1β concentration was greater in ulcer group ; TNF-α showed no difference between groups; ICAM-1 was greater in patients with ulcers; IL-10 was no different between groups.

Plasma concentrations of the proinflammatory cytokine IL1-β (pg/mL) (ss_u_ vs. ss_n_ median(IQR): 0.34 (1.28) vs. 0 (0.08); *p* = 0.0178), but not TNF-α (pg/mL) (1.99 (4.3) vs. 0.97 (3.38) was significantly greater in subjects with ulcers. Furthermore, comparing patients with leg ulcers with patients without ulcers, sICAM-1 (ng/mL) (141.8 (257.41) vs. 0.41 (107.3); *p* = 0.0152), but not IL-10 (ng/mL) (11.01 (15.95) vs. (2.98 (11.92) was significantly greater in the ulcer group (Figure 1).

**Figure 2:**
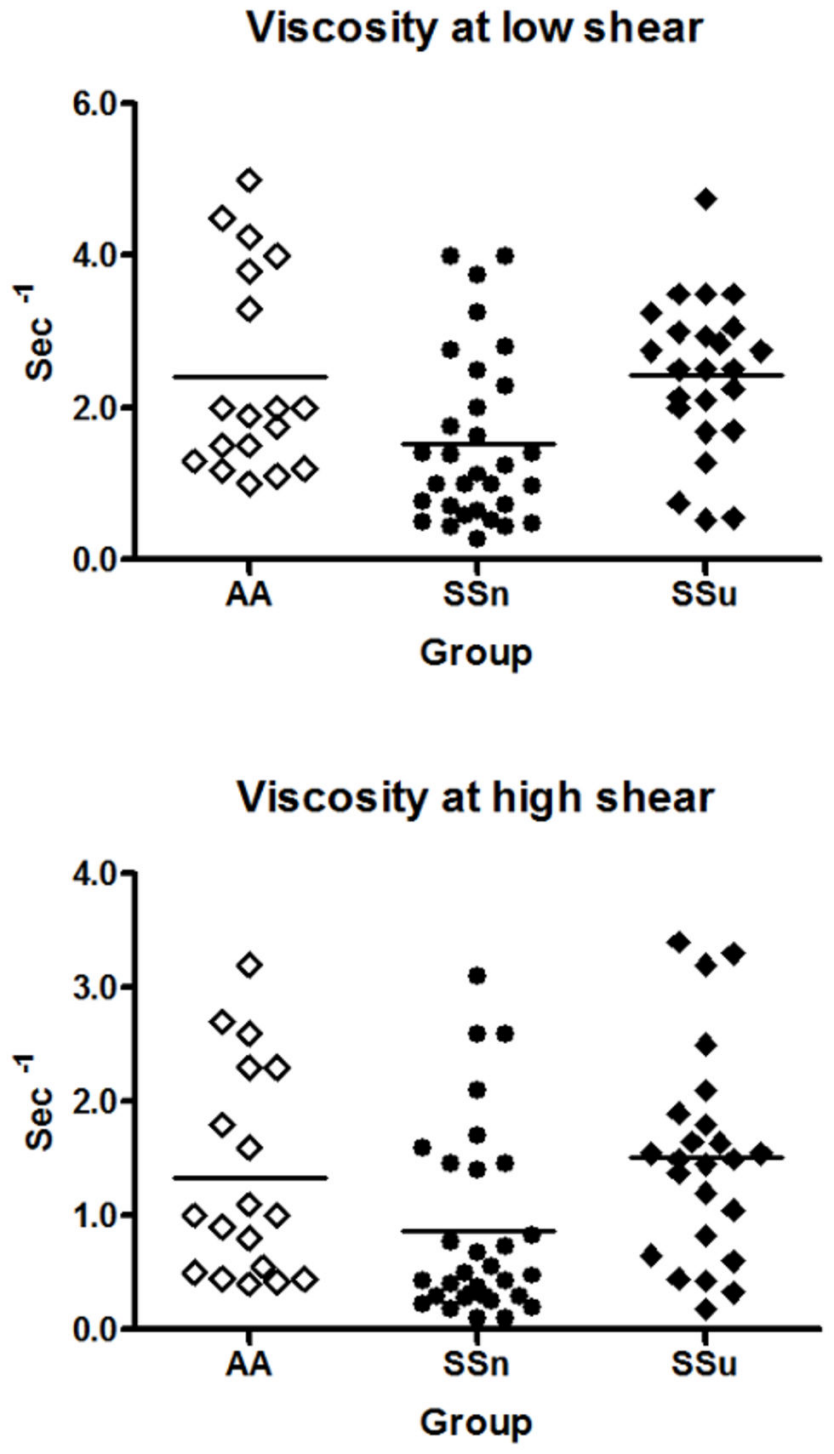
Whole blood viscosity distribution at low and high shear rates in SCD patients with ulcers and those without. WBV was greater in the group with ulcers at both the low and high shear rates. Both groups had outliers above and below their respective WBV ranges.

The distribution of WBV was skewed and was normalized by Napier logarithmic transformation. WBV in the SS_u_ group at 46 sec^-1^ and at 230 sec^-1^ was 1.9 (95%CI 1.2, 3.1) (p<0.04) and 2.3 (95%CI 1.2, 4.4) (*p*<0.007) times greater than the SS_n_ group respectively (Figure 2).

**Figure 3:**
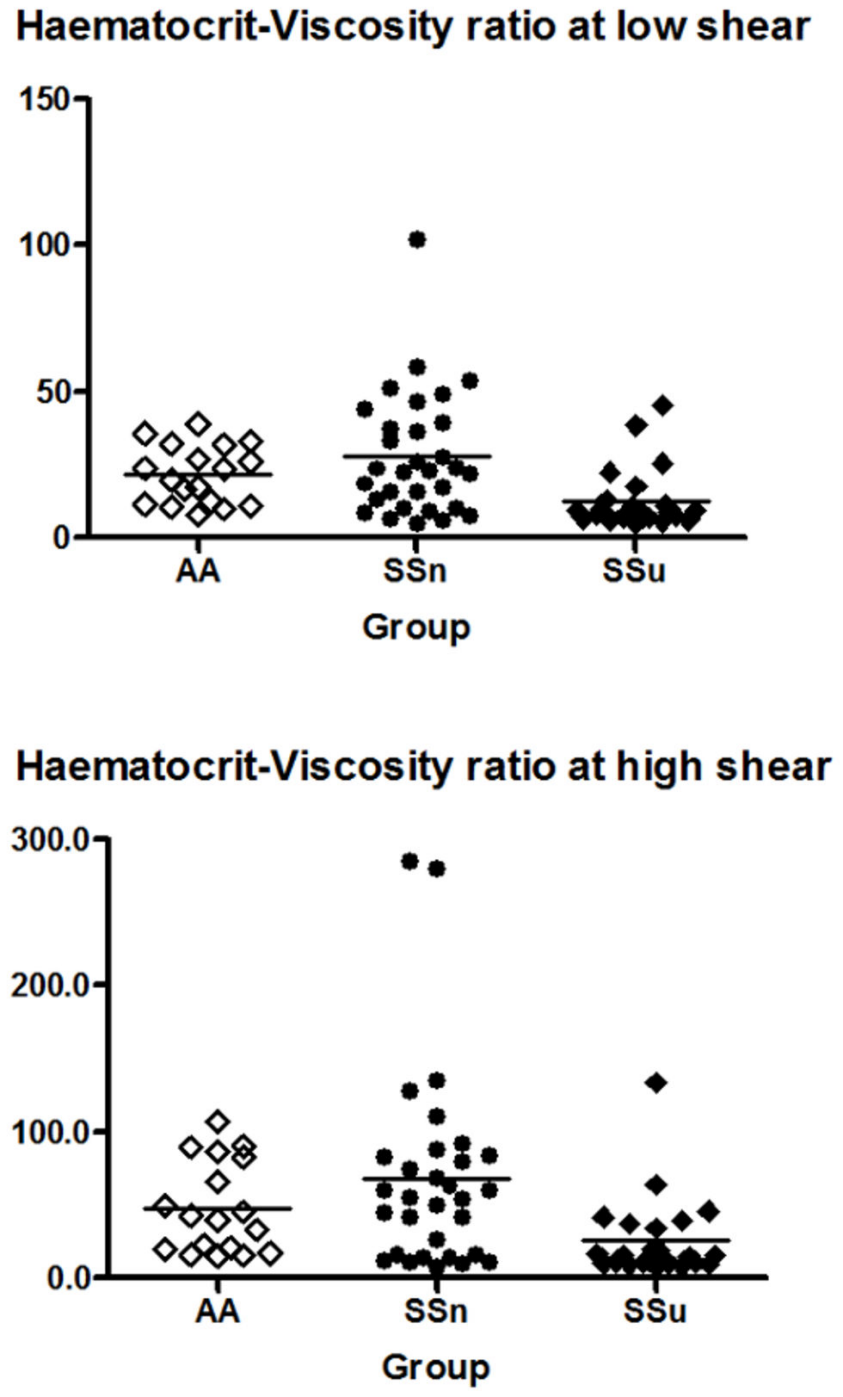
Erythrocyte transport effectiveness at low and high shear rates in SCD patients with ulcers and those without. HVR was lower in the group with ulcers at both low and high shear rates of WBV.

The haematocrit–viscosity ratio was significantly lower in sickle cell disease subjects with ulcers in comparison to the non-ulcer group at 46 sec^-1^ (SS_u_ vs. SS_n_: 8.73; 4.89, 45.47 vs. 22.85; 4.75, 101.78; *p* =0.011) and 230 sec^-1^ (SS_u_ vs. SS_n_: 15.08; 7.4, 133.89 vs. 54.58; 7.96, 285; *p* =0.011), respectively. In SS_u_ subjects the HVR was less than half that of the SS_n_ subjects (Figure 3). There was a significant shear-dependent relationship between BMI and HVR. At low shear rate there was a -2.89 (95% CI; -0.003, 0.146; *p* = 0.015) change in the HVR with each unit increase in BMI. However, at high shear rate there was no significant association with the HVR and 1 unit change of BMI.

**Figure 4:**
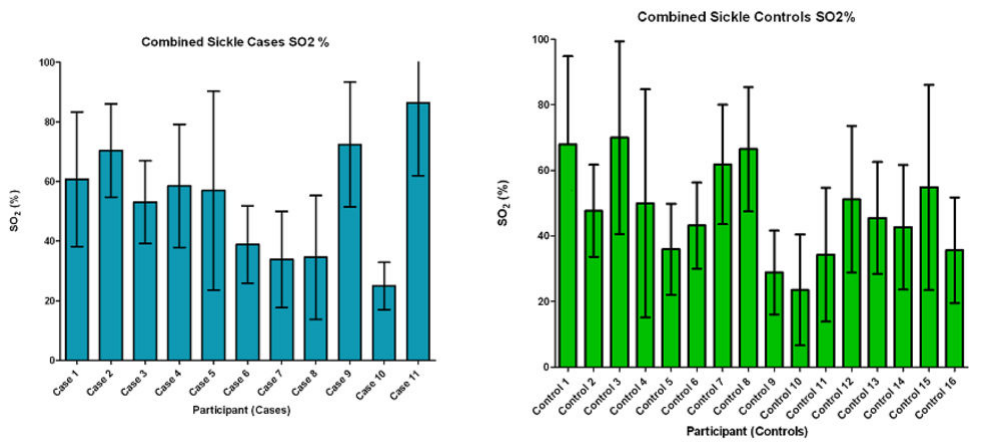
Lightguide haemoglobin oxygen saturation observations in SCD subjects with active leg ulcers (cases) and controls. Pooled mean SO_2_ results taken at different sites along the length of the lower leg. Data are median with interquartile range.

**Figure 5:**
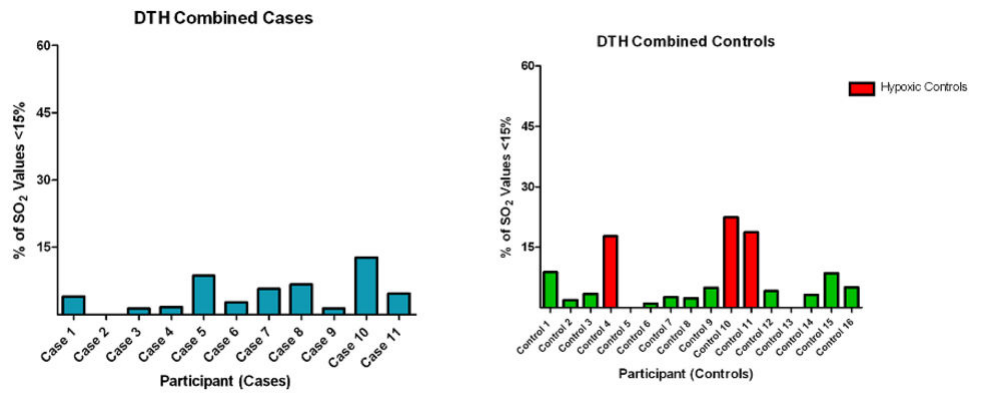
Degree of tissue oxygen saturation distributions in SCD cases and controls. Figure shows percentage of observations with SO_2_ values below 15%.

There were no differences in cutaneous microvascular oxygen saturation as determined by lightguide spectrophotometry between SS_n_ and SS_u_ (Figure 4). Mean oxygen saturation was lower in subjects with ulcers than SS controls (mean +/- SD SO_2_: 45.02±12.97 versus 50.02±16.49). Both groups occupied similar SO_2_ ranges of 25-72.16 and 22-75.69 in cases and controls, respectively (Figure 4). However, none of the 11 subjects with active ulcers were classified as having hypoxia in the lower leg compared with 3 in the control group (Figure 5). Furthermore, SO_2_ were similar in the same subject from one leg to the next. There were no apparent relationships between the lightguide measurements and any of the investigated mediators of disease severity.

## Discussion

These data support the hypothesis that abnormal rheology, inflammation and endothelial dysfunction may be associated with chronic leg ulceration in sickle cell disease. Thus we found that sICAM-1 and IL-1β, markers of endothelial function and inflammation respectively, were significantly greater in SS_u_ vs. SS_n_. Furthermore, consistent with previous reports suggesting an up-regulation of inflammatory pathways in sickle cell disease vs. controls, we found significantly greater concentrations of the inflammatory cytokine TNF-α [10,33–36], but not IL-1β or the adhesion molecule, ICAM-1 in the sickle cell disease group. There were no differences in the degree of tissue hypoxia between the sickle cell disease groups as measured by Visible Lightguide spectrophotometry.

Belcher et al. reported that and IL-1β serve as a marker of monocyte activation and that monocytosis is a common feature of sickle cell disease [10]. In addition, IL-1β is suggested to be involved in the activation of endothelial cells to an inflammatory phenotype. Endothelial adhesion enhances sickle cell polymerization by delaying the transit of red cells through micro-vessels, thereby promoting hypoxia and tissue infarction. These processes are amplified in the presence of the inflammatory cytokine IL-1β, which serve as a sort of triggering mechanism wherein the endothelium assumes an inflammatory state following a series of molecular conformational alterations. Shiu et al. demonstrated an increase in both membrane bound and soluble sICAM-1 expression upon perfusion of endothelial cells with sickle erythrocytes [13]. This marked an important finding since activation of the endothelium was brought about by red cells alone, not components of the plasma. Sickle cell disease subjects have been shown to be unusually susceptible to a variety of infections possibly as a consequence of reticuloendothelial dysfunction [33,37–40]. In light of such observations and the comparability of IL-10 concentration between SS_n_ and SS_u_ in the present study, it seems possible that there may be an imbalance between the expression of inflammatory and anti-inflammatory immune responses in sickle cell disease subjects with chronic leg ulcers.

Earlier *in vitro* studies have indicated that a molecular switch from a pro-inflammatory to anti-inflammatory phenotype is critical for the resolution of inflammation [25,41]. Inefficient inhibition of pro-inflammatory pathways could explain the chronic inflammatory phenotype of SCD and could be partly responsible for causing a predisposition for ulceration in a subset of SCD patients. An intriguing observation from the present findings was the discrepancy in inflammatory cytokine concentrations between the SCD patient groups, reported as significantly greater IL-1β but not TNF-α in SS_u_ vs. SS_n_. Both TNF-α and IL-1β have a common activation pathway by way of nuclear factor kappa-beta (NFk-β) translocation [10]. However, since TNF-α and IL-1β have independent post-translational regulation mechanisms and endogenous inhibitors, these could account for differences in serum cytokine concentrations [42–45]. Therefore, treatment regimens targeted at reducing specific pro-inflammatory mediators involved in local inflammation in SCD may be useful in the management of sickle leg ulcers.

The involvement of the pro-inflammatory cytokines IL-1β and TNF-α in promoting endothelial adhesiveness, leukocyte activation [46] and the coagulation cascade [47] could render them potent mediators of episodic vasoocclusion in sickle cell disease and in promoting chronic leg ulcers. The properties of whole blood are such that changes in the whole blood viscosity have an inverse effect on the rate of blood flow. *In vitro*, this has important implications as normal blood flow can always be interrupted by any or a combination of factors which affect either shear rate or viscosity. In sickle cell blood, the abnormal rheological properties conferred by the polymerization dependent rigidification of the erythrocyte membrane are worsened by small increments in WBV. Cooperative vasoocclusion may result from clumping of rigid red cells and leucocytes and their adherence to the activated endothelium. The greater concentrations of sICAM-1, IL-1β, as wells as the raised WBV in SS_u_ compared with SS_n_ subjects may be indicative of inflammatory mediated vaso-occlusion in the pathogenesis of sickle cell leg ulcers. While it is conceivable how the up-regulation of adhesion factors may adversely affect WBV *in vivo*, it is unclear how these associations may be relevant *in vitro* since there is no endothelium for RBC adherence.

The present findings showed increased mean cell size in the ulcer group compared with HbSS controls. Meanwhile, RDW was similar between groups, suggestive of similar cell densities and therefore unlikely to be associated with the higher WBV in the ulcer patients. Acute phase increases in certain proteins with attendant decrease in haematocrit have been reported in diabetic foot ulcers [48] and arteriosclerosis obliterans [49]. Further investigations into the association between acute phase proteins and plasma viscosity among these ulcer patients may aid in explaining our WBV findings. It is possible that a significantly greater mean cell volume, although within normal limits, could be associated with greater WBV in patients with ulcers compared with those without ulcers. A lower HVR or otherwise termed ‘erythrocyte transport effectiveness’ [50,51] [31] describes the rheological potential of the blood and therefore serves an index for assessing the likelihood of oxygen reaching tissues in the microcirculation for a given haematocrit and WBV. However, oxygen delivery is a complex process and is ultimately determined by several other factors including pH, temperature and red cell 2,3-diphosphoglycerate concentration.

The viscosity recordings in the present work were lower than reported for normal or sickle blood [28,31]. The lower values may be due to a systematic error in the cone-plate viscometer used in our study.

There are no reports in the literature regarding the involvement of body mass index (BMI) in sickle leg ulceration. However, we reported a significantly greater BMI in patients with ulcers than those without ulcers which was related a significantly greater mean weight in the ulcer group. Earlier works have suggested an association between ulceration and BMI in diabetics [52,53]. Sickle cell disease ulceration could share a common aetiology since both conditions show similarities in several vascular complications, notably retinopathy and leg ulceration [52,54,55]. However, reports among diabetics are conflicting with observations of both positive [52] and negative [53] associations between BMI and ulceration. It is also possible that the greater BMI in the present study is related to the higher mean age in the ulcer group. The age difference between the ulcer group and patients without ulcers is consistent with findings of the role of advancing age in ulceration [4,56]. However, it is unclear how this age discrepancy may influence haemorheological determinations across groups and between genotypes.

The Lightguide flow data indicated that microvascular oxygen saturation was not a precipitating factor in leg ulceration since there was no difference in the degree of tissue oxygenation in subjects with ulcers and those without. These data as determined by our definition for hypoxia appear conflicting in consideration of the lesser HVR in subjects with ulcers. However, whilst the HVR describes the efficiency of oxygen transport by RBC, it does not quantify local tissue perfusion in absolute terms. The mean SO_2_ values recorded along the length of the lower leg were lesser in subjects with ulcers for both the right and left leg measurements. It is likely that local hypoxia alone is not a strong indicator for the development and/or progression of leg ulcers in SCD. Mechanical injury to the endothelium by trapped rigid cells, increased number of leucocytes leading to chronic inflammation and vascular dysfunction could represent more important biomarkers in sickle cell leg ulceration. Studies have shown that the proposed ‘fibrin cuff’ in venous diseases do not cause a significant difference in the observed diffusion block to flowing blood between controls and subjects to implicate hypoxia in its aetiology [57]. Trapped leucocytes (by way of larger size and rigidity) in the lower leg could be a stimulus for ulceration by their damaging effects on connective tissue, cell membrane and the endothelium. Paradoxically, some authors believe that WBC in the interstitium may be targeted at fibroblasts where they promote increased cellular proliferation and fibrotic connective tissue growth and the characteristic thickened hyperpigmented skin associated with foot ulcers [57]. Furthermore, histological evidence has indicated the infiltration of the capillaries of the papillary plexus by inflammatory mediators such as monocytes, macrophages and fibrin.

Other reports have likened chronic leg ulcers to a sickle cell disease sub-phenotype characterized by chronic hyper-haemolysis and a significantly lowered haemoglobin and significantly increased lactate dehydrogenase levels [4,9,58]. These contrast the present findings where we observed no differences in these variables between the ulcer group and patients without ulcers. The reasons for these differences are not clear, especially regarding conflicts among findings within the Jamaican population [4]. However, these observations suggest the presence of leg ulcers in these patients may not always be associated with more severe haemolysis than patients without ulcers. Similarities observed here between SS_n_ and SS_u_ could also be due to the high variation in LDH values from one patient to the next as demonstrated by wide sample dispersion.

In conclusion, we have reported that sickle cell disease is associated with an up-regulation of inflammatory and adhesive plasmatic components. There does not appear to be a direct link between microvascular oxygen saturation and ulceration as determined by spectrophotometry. On the other hand, the HVR suggests that subjects with ulcers have a greater rheological deficit than those without, namely, lower haematocrit but a higher whole blood viscosity. While endothelial dysfunction and increased whole blood viscosity in ulcer patients could simply represent consequences of localized inflammation resulting from the ulcer scar and not a cause of ulceration, the complexity of SCD vasculopathy leaves much to be understood. Another possibility is that in predisposed patients, vaso-occlusion induced inflammation could lead to vascular damage, increased inflammation, endothelial activation and ischaemia-reperfusion injury. Prolonged inflammatory responses and reduced erythrocyte transport effectiveness could therefore be important in inciting a pro-inflammatory micro-environment. Therefore, an elevation in the concentrations of pro-inflammatory cytokines and adhesion molecules with concomitant increase in whole blood viscosity may play a role in the pathogenesis of leg ulceration in sickle cell disease.
